# Tumor Budding and E‐Cadherin Loss as Robust Prognostic Markers in Pancreatic Ductal Adenocarcinoma: A Study in a Turkish Patient Cohort

**DOI:** 10.1155/cjgh/9097621

**Published:** 2025-11-14

**Authors:** Tevhide Bilgen Özcan, Esra Pasaoglu, Osman Bilgin Gülçiçek

**Affiliations:** ^1^ Department of Medicine Pathology, Bagcilar Education and Research Hospital, University of Health Sciences, Istanbul, Türkiye, akdeniz.edu.tr; ^2^ Department of General Surgery, Bagcilar Education and Research Hospital, University of Health Sciences, Istanbul, Türkiye, akdeniz.edu.tr

**Keywords:** E-cadherin, pancreatic ductal adenocarcinoma, prognostic biomarker, tumor budding

## Abstract

**Objective:**

This study investigates the prognostic significance of tumor budding and its association with E‐cadherin expression in pancreatic ductal adenocarcinoma (PDAC) with a focus on a Turkish patient cohort.

**Methods:**

A total of 76 patients who had undergone resection of PDAC were analyzed. Tumor budding was assessed according to Tumor Budding Consensus Conference (ITBCC) guideline and classified as low (1–4 buds), medium (5–9 buds), or high (≥ 10 buds). E‐cadherin expression was assessed by immunohistochemistry and categorized as low, moderate, or high. The correlations between tumor budding, clinicopathological factors, and survival were statistically analyzed and examined, and *p* < 0.05 was considered significant.

**Results:**

Tumor budding was detected in 42.1% of patients, significantly associated with advanced tumor stage (*p* < 0.001), perineural invasion (*p* = 0.015), and lymphatic invasion (*p* = 0.005). Reduced E‐cadherin expression was strongly correlated with tumor budding, which occurred in 83.3% of patients with weak (+) expression. Kaplan–Meier survival analysis demonstrated a median survival of 7.03 months in patients with tumor budding, compared to 21.7 months in those without tumor budding (*p* < 0.001). Multivariate analysis confirmed tumor budding as an independent prognostic factor (HR = 6.594, 95% CI: 1.825–23.818, *p* = 0.004).

**Conclusions:**

Tumor budding is a reliable prognostic marker in PDAC, demonstrating significant associations with poor clinical outcomes, including advanced tumor stage and reduced survival. These findings support the integration of tumor budding into clinical guidelines to enhance prognostic precision and guide treatment decision‐making.

## 1. Introduction

Pancreatic ductal adenocarcinoma (PDAC) is a highly aggressive malignancy that causes significantly to cancer‐related mortality worldwide [1]. Although it accounts for only 3% of all cancers diagnosed, PDAC is one of the leading causes of cancer‐related death with a five‐year survival rate of only 11% [2]. The aggressiveness of this disease, its resistance to therapy, and its tendency to metastasize early contribute to its poor prognosis. Although surgical resection is the only option for cure, even patients with resectable PDAC face high recurrence rates and limited long‐term survival [3]. This highlights the urgent need for novel prognostic biomarkers to improve disease progression prediction and guide therapeutic strategies.

One such novel marker is tumor budding, a histopathological phenomenon characterized by the presence of isolated single tumor cells or small clusters (typically less than five cells) at the invasive front of the tumor [4]. Tumor budding is considered an indicator of increased tumor aggressiveness as it reflects the ability of cancer cells to detach from the primary tumor, invade surrounding tissue, and metastasize to distant sites [5]. The prognostic significance of tumor budding has been extensively studied in colorectal cancer and has now been incorporated into standardized reporting systems such as the International Tumor Budding Consensus Conference (ITBCC) guidelines. There are also studies in the literature investigating tumor budding in lung cancer, and it has been reported to be associated with poor prognosis [6, 7]. However, research on tumor budding in PDAC is still relatively limited [8]. Existing studies suggest that tumor budding is associated with worse clinical outcomes, including shorter progression‐free and overall survival, but standardized methods for the evaluation and grading of tumor budding in pancreatic tumors are still lacking [9]. In addition, the biological basis of tumor budding in PDAC, particularly its relationship to molecular markers such as E‐cadherin, is still poorly understood [10]. E‐cadherin, a key component of adherens junctions, plays a central role in maintaining the integrity of epithelial cells, and the loss of E‐cadherin is a key feature of the epithelial‐to‐mesenchymal transition (EMT) [11, 12]. In PDAC, loss of E‐cadherin expression has been associated with aggressive tumor behavior and poor treatment outcomes [13]. EMT is thought to play a key role in disease progression and the development of drug resistance in PDAC, while tumor budding reflects processes in which cells adopt a mesenchymal phenotype that increases their ability to migrate, invade, and resist apoptosis [14].

This study aims to address these gaps by evaluating the prognostic impact of tumor budding in a cohort of Turkish patients with PDAC. Additionally, it investigates the relationship between tumor budding and E‐cadherin expression, assessing their combined contribution to clinical outcomes. By integrating histopathological and molecular data, this study seeks to improve prognostic accuracy, enhance understanding of PDAC progression mechanisms, and ultimately contribute to better treatment strategies and patient outcomes.

## 2. Materials and Methods

This study conformed to the tenets of the Declaration of Helsinki and was approved by the institution’s ethics committee. Informed consent was obtained from all participants prior to collection and analysis of samples and clinical data.

### 2.1. Study Design and Patient Selection

A total of 76 patients who underwent surgical resection for PDAC at a single center between 2017 and 2024 were included in this study. Clinical and pathological data, including age, gender, tumor location, and disease stage, were obtained from the medical records. Cases with missing clinical data or insufficient tissue samples were excluded from the analysis.

### 2.2. Histopathologic Assessment

Tumor budding was defined as the presence of isolated tumor cells or small clusters comprising fewer than five cells at the invasive margin of the tumor. Hematoxylin and eosin (H&E)‐stained slides were independently reviewed by two experienced pathologists who were blinded to the clinical outcomes. Tumor budding was evaluated in hotspot regions under high‐power field (HPF, 40x magnification) and categorized into three groups: low (0–4 buds), intermediate (5–9 buds), and high (≥ 10 buds).

To ensure accuracy and standardization, tumor budding was assessed using two complementary methods:

1. HPF method (40x magnification): Tumor buds, defined as single tumor cells or clusters of fewer than five cells at the invasive tumor front, were counted in three separate hotspot regions within a field area of 0.237 mm^2^ under 40x magnification.
2. ITBCC method (20x magnification): Tumor budding was evaluated following ITBCC guidelines, where tumor buds were quantified within a single hotspot region with an area of 0.785 mm^2^ under 20x magnification.

The results obtained from both methods were analyzed to ensure a robust, standardized, and reproducible assessment of tumor budding.

### 2.3. Immunohistochemistry

To evaluate tumor budding, 4‐μm sections were prepared from 10% buffered formalin‐fixed, paraffin‐embedded tumor tissue blocks. These sections were stained using anti‐cytokeratin (CK) CK8/18 antibodies. Diaminobenzidine (DAB) was used as the chromogen, while hematoxylin served as the nuclear counterstain. Positive control tissues were included alongside the study samples for validation purposes.

E‐cadherin expression was assessed through immunohistochemistry on formalin‐fixed, paraffin‐embedded tissue sections. Four‐micrometer sections mounted on poly‐L‐lysine‐coated slides underwent deparaffinization, rehydration, and antigen retrieval in citrate buffer (pH 6.0) using microwave heating. Endogenous peroxidase activity was blocked using 3% hydrogen peroxide. The sections were then incubated overnight at 4°C with a primary anti‐E‐cadherin monoclonal antibody (1:200 dilution), followed by treatment with an HRP‐conjugated secondary antibody and DAB chromogen. Hematoxylin was used for nuclear counterstaining.

E‐cadherin expression was evaluated semiquantitatively based on the percentage and intensity of staining in tumor cells. Cases were classified based on staining patterns, with normal expression defined as strong membranous staining observed in 70% or more of the cells, and reduced expression characterized by weak or absent membranous staining.

### 2.4. Statistical Analysis

The Pearson chi‐square test was utilized to assess differences between categorical variables. When any cell in a 2 × 2 contingency table had an expected frequency below 5, Fisher’s exact test was employed. For contingency tables larger than 2 × 2 with low expected cell counts, the Monte Carlo simulation method was used to derive reliable *p* values. A *p* value of less than 0.05 was considered indicative of statistical significance. In cases where expected frequencies ranged between 1 and 5 and the total sample size was 40 or more, the continuity‐corrected chi‐square test (Yates’ correction) was applied. If expected frequencies were below 1, the sample size was less than 40, or any cell contained zero observations, Fisher’s exact test was performed. Survival analyses were performed using univariate and multivariate Cox proportional hazards regression models to assess the impact of tumor budding on overall survival. Hazard ratios (HRs) and their 95% confidence intervals (CIs) were calculated. Kaplan–Meier survival curves were constructed, and differences between categories of budding were compared using the log‐rank test. A *p* value of < 0.05 was considered statistically significant. All statistical analyses were performed using SPSS software version 30.0 (IBM Corp., Armonk, NY, USA).

## 3. Results

This study analyzed the clinical parameters of 76 patients with pancreatic cancer. Regarding age distribution, 43.4% (33/76) of the patients were under 60 years old, while 56.6% (43/76) were over 60 years old. In terms of gender distribution, 53.9% (41/76) were male, and 46.1% (35/76) were female. The anatomical location of the tumor was most commonly in the head of the pancreas (76.3%, 58/76), followed by the tail (10.5%, 8/76), the corpus (5.3%, 4/76), and the uncinate region (2.6%, 2/76). Perineural invasion was observed in 85.5% (65/76) of cases, and lymphovascular invasion in 88.2% (67/76). According to the T stage distribution, 25% (19/76) of the patients were at tumor stage I, 63.2% (48/76) at tumor stage III, and 7.9% (6/76) at tumor stage IV. Based on the node stage, 17.1% (13/76) were classified as node stage 0, 34.2% (26/76) as node I, and 48.7% (37/76) as node stage II. In terms of prognostic stage distribution, 3.9% (3/76) were in stage IA, 6.6% (5/76) in stage IB, 6.6% (5/76) in stage IIA, 34.2% (26/76) in stage IIB, and 48.7% (37/76) in stage III. Positive surgical margins were identified in 22.4% (17/76) of the cases. Regarding differentiation grade, 34.2% (26/76) of the tumors were highly differentiated, 44.7% (34/76) moderately differentiated, and 21.1% (16/76) poorly differentiated. At the final follow‐up, 63.2% (48/76) of the patients had deceased, while 36.8% (28/76) were still alive and undergoing treatment.

**Figure 1:**
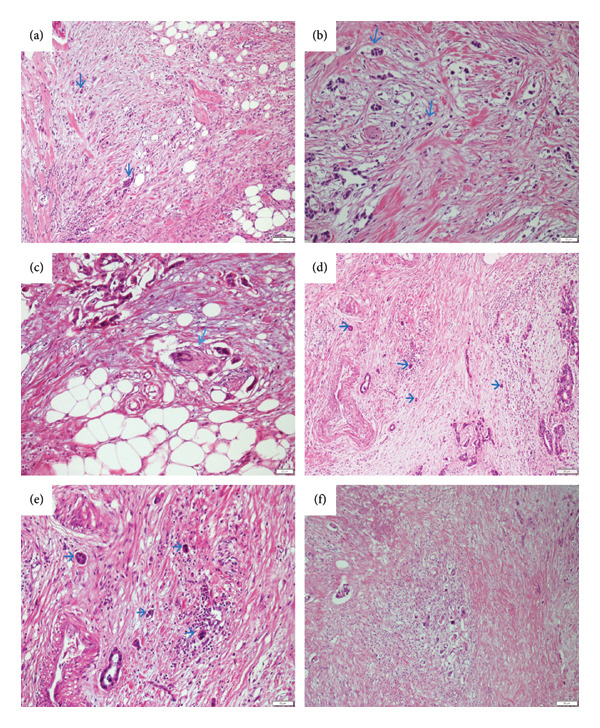
Microscopic images of tumor budding stained with hematoxylin and eosin (tumor budding levels: low: 1–4 buds, moderate: 5–9 buds, and high: > 10 buds): (a) tumor invasive margin showing low and high levels of tumor budding indicated by arrows (10x); (b) tumor invasive margin showing low and high levels of tumor budding indicated by arrows (20x); (c) perineural invasion of low and moderate levels of tumor budding indicated by arrows and tumor budding at the tumor invasive margin (20x); (d) tumor invasive margin showing low and high levels of tumor budding indicated by arrows (10x); (e) tumor invasive margin showing low and high levels of tumor budding indicated by arrows (20x); (f) tumor invasive margin showing low and high levels of tumor budding indicated by arrows (10x).

**Figure 2:**
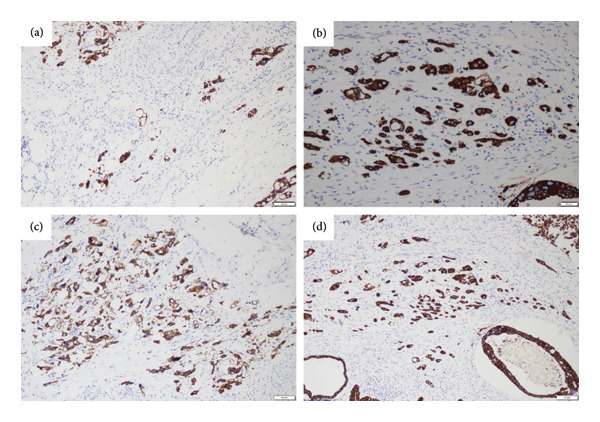
Microscopic images of numerous tumor buddings positively stained with CK8/18 by immunohistochemistry (a–d).

H&E tumor budding statuses are shown in Figure 1, and numerous tumor buddings stained positive with CK8/18 are shown in Figure 2. Tumor budding was defined as low if there were 1–4 buds, medium if there were 5–9, and high if there were more than 10. Tumor budding was found positive in 42.1% (32/76). Perineural and lymphovascular invasion was observed in all patients with tumor budding.

**Figure 3:**
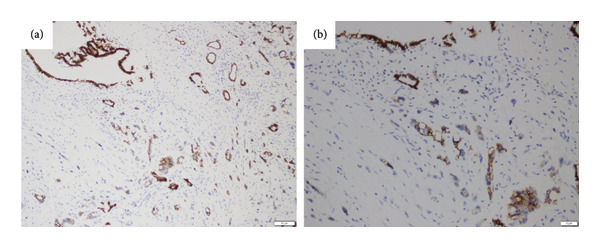
Microscopic images showing the loss of membranous staining in tumor buddings with E‐cadherin by immunohistochemistry: (a) 10x; (b) 20x.

The images showing the loss of membranous staining in tumor buddings with E‐Cadherin are presented in Figure 3. In terms of E‐Cadherin expression patterns, 23.7% (18/76) were reported as weak (+), 40.8% (31/76) as moderate (++), and 35.5% (27/76) as high (+++). When examining E‐cadherin expression status, tumor budding was observed in 83.3% (15/18) of patients with weak (+) expression patterns, 41.9% (13/31) of patients with moderate (++) expression patterns, and 14.8% (4/27) of patients with high (+++) expression patterns.

The relationship between tumor budding and patients’ clinical parameters was evaluated using the Pearson chi‐square test. When expected cell counts were below 5 in any 2 × 2 contingency table, Fisher’s exact test was applied. For larger tables with low expected frequencies, the Monte Carlo simulation method was employed to obtain reliable *p* values. The findings are summarized in Table 1. No significant associations were found between tumor budding and age, gender, or anatomical tumor location (*p* > 0.05). However, statistically significant relationships were identified between tumor budding and perineural invasion (*p* = 0.015), lymphovascular invasion (*p* = 0.005), T stage (*p* < 0.001), N stage (*p* < 0.001), prognostic stage (*p* < 0.001), surgical margin positivity (*p* = 0.003), differentiation grade (*p* < 0.001), and E‐cadherin pattern (*p* < 0.001). Specifically, tumor budding positivity was strongly associated with poor prognostic indicators such as perineural and lymphovascular invasion, advanced tumor stage, nodal Stage II, and prognostic Stage III. Furthermore, low differentiation and high E‐cadherin patterns (+++) were markedly higher in cases with tumor budding positivity.

**Table 1:** Analysis of clinical parameters versus tumor budding positivity of patients.

Parameters		Tumor budding		*p* value
		Negative	Positive	
Age	< 60	21 (63.6)	12 (36.40)	0.257
	> 60	23 (53.5)	20 (46.5)	

Gender	Male	24 (58.5)	17 (41.5)	0.544
	Female	20 (57.1)	15 (42.9)	

Tumor localization	Head	38 (65.5)	20 (34.5)	0.062
	Tail	2 (25.0)	6 (75.5)	
	Corpus	0	4 (100)	
	Uncinate	1 (50.0)	1 (50.0)	
	Head and uncinate	1 (50.0)	1 (50.0)	
	Head and corpus	1 (100.0)	0	
	Corpus and neck	1 (100.0)	0	

Perineural invasion	Negative	10 (90.9)	1 (9.1)	0.015^∗^
	Positive	34 (52.3)	31 (47.7)	

Lymphovascular invasion	Negative	9 (100.0)	0	0.005^∗^
	Positive	35 (52.2)	32 (47.8)	

Tumor stage	I	3 (100.0)	0	< 0.001^∗∗^
	II	17 (89.5)	2 (10.5)	
	III	24 (50.0)	24 (50.0)	
	IV	0	6 (100.0)	

Nodal stage	0	13 (100.0)	0	< 0.001^∗∗^
	I	22 (84.6)	4 (10.9)	
	II	9 (24.3)	28 (75.7)	

Prognostic stage	IA	3 (100.0)	0	< 0.001^∗∗^
	IB	5 (100.0)	0	
	IIA	5 (100.0)	0	
	IIB	22 (84.6)	4 (15.4)	
	III	9 (24.3)	28 (75.7)	

Surgical margin	Negative	40 (67.8)	19 (32.2)	0.003^∗^
	Positive	4 (23.5)	13 (76.5)	

Degree of differentiation	High	25 (96.2)	1 (3.8)	< 0.001^∗∗^
	Medium	16 (47.1)	18 (52.9)	
	Low	3 (18.8)	13 (81.3)	

E‐cadherin pattern	Low (+)	3 (16.7)	15 (83.3)	< 0.001^∗∗^
	Medium (++)	18 (58.1)	13 (41.9)	
	High (+++)	23 (85.2)	4 (14.8)	

^∗^ *p* < 0.05 is statistically significant.

^∗∗^ *p* < 0.001 is statistically significant.

The relationship between survival time and clinical parameters in pancreatic cancer patients was analyzed using Kaplan–Meier analysis and is presented in Figure 4 and Table 2. The shortest survival times were observed in the corpus (5.08 months, 95% CI: 0.2–10.704) and tail (8.33 months, 95% CI: 0.84–15.82) regions of the pancreas, while the longest survival times were noted in tumors localized in the head (15.55 months, 95% CI: 11.43–19.63) and uncinate (14.65 months, 95% CI: 0.65–28.66) regions. For patients with positive lymphovascular invasion, the median survival time was 13.20 months (95% CI, 10.03–16.37), compared to 39.87 months (95% CI, 4.46–75.29) for those without invasion. Regarding tumor stage, the median survival time was 15.97 months (95% CI, 14.36–17.57) for stage II, 9.80 months (95% CI, 2.75–16.85) for stage III, and 4.27 months (95% CI, 0.00–10.27) for stage IV. In terms of lymph node involvement, patients with stage II had a median survival time of 7.50 months (95% CI, 5.08–9.91), while those with stage I had 19.10 months (95% CI, 11.21–26.94). For prognostic stages, the median survival time was 29.65 months (95% CI, 14.26–45.03) for stage IIA, 19.03 months (95% CI, 13.45–24.62) for stage IIB, and 9.61 months (95% CI, 6.29–12.93) for stage III. The median survival time for patients with positive surgical margins was 7.03 months (95% CI, 2.65–11.41), compared to 15.07 months (95% CI, 12.57–17.56) for those with negative margins. For degree of differentiation, patients with low‐grade tumors had a median survival time of 7.27 months (95% CI, 5.24–9.29), compared to 21.80 months (95% CI, 18.29–25.31) for those with high‐grade tumors. Regarding E‐cadherin patterns, the median survival time for patients with the high (+++) pattern was 28.20 months (95% CI, 14.46–41.94), while those with the + pattern had 7.03 months (95% CI, 1.13–13.27). Lastly, patients with tumor budding had a median survival time of 7.03 months (95% CI, 1.95–12.12), compared to 21.70 months (95% CI, 17.66–25.73) for those without budding. Age, gender, and perineural invasion were found to have no significant impact on survival (*p* > 0.05).

Figure 4Kaplan–Meier survival curves depicting cumulative survival probabilities for patients stratified by clinical parameters: (a) tumor budding; (b) age; (c) gender; (d) tumor location; (e) perineural invasion; (f) lymphovascular invasion; (g) T stage; (h) N stage; (i) prognostic stage; (j) surgical margin; (k) degree of differentiation; (l) E‐cadherin pattern.

**Figure:**
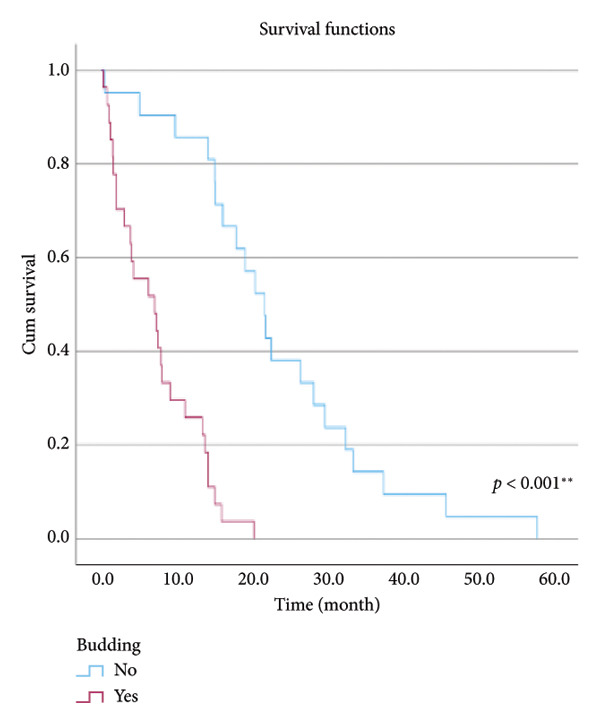
(a)

**Figure:**
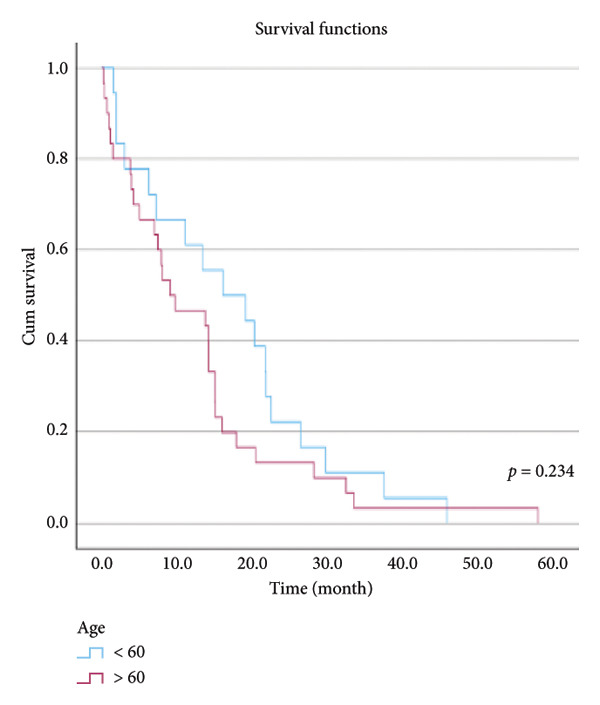
(b)

**Figure:**
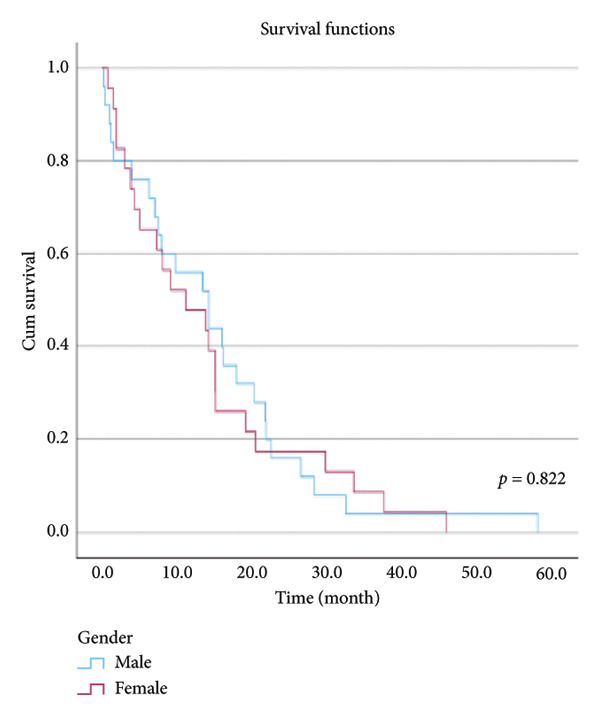
(c)

**Figure:**
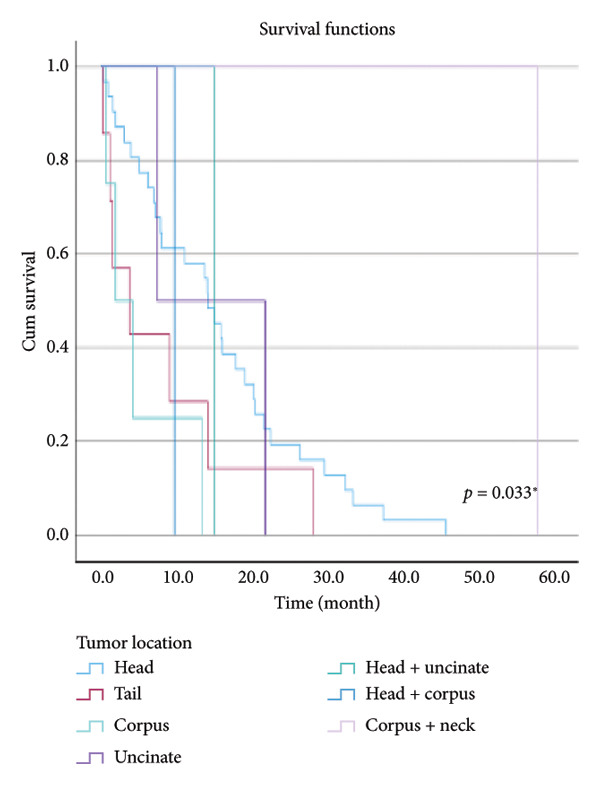
(d)

**Figure:**
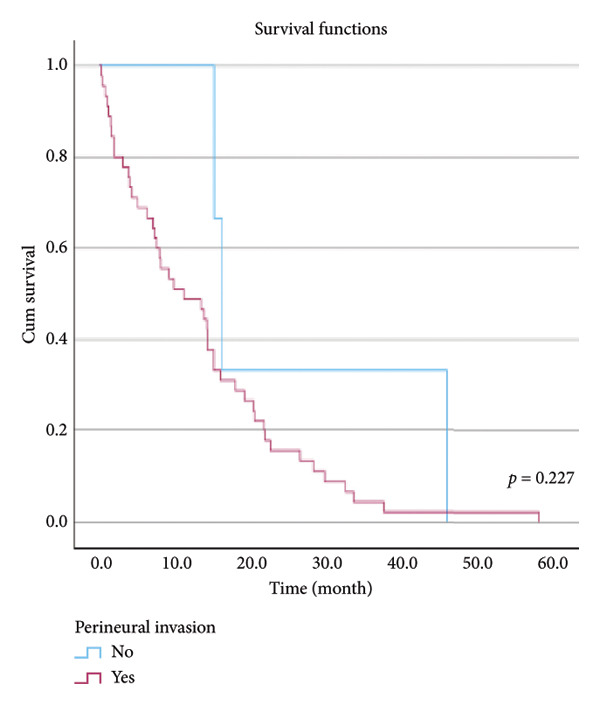
(e)

**Figure:**
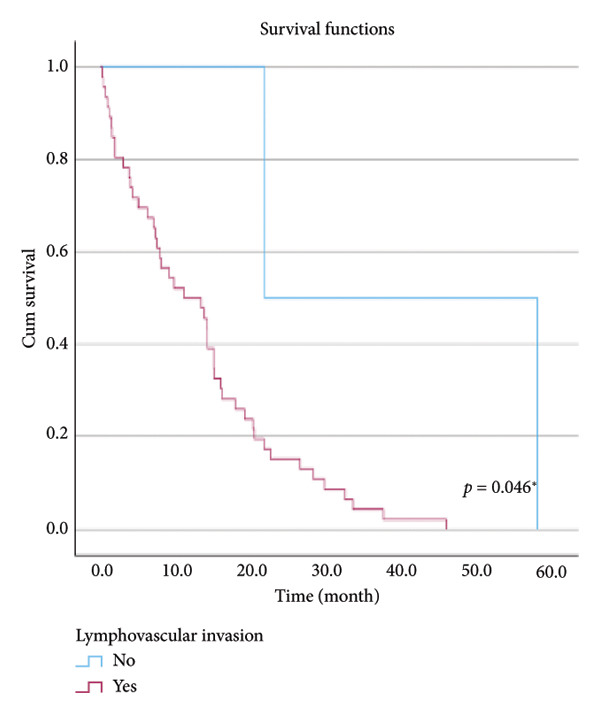
(f)

**Figure:**
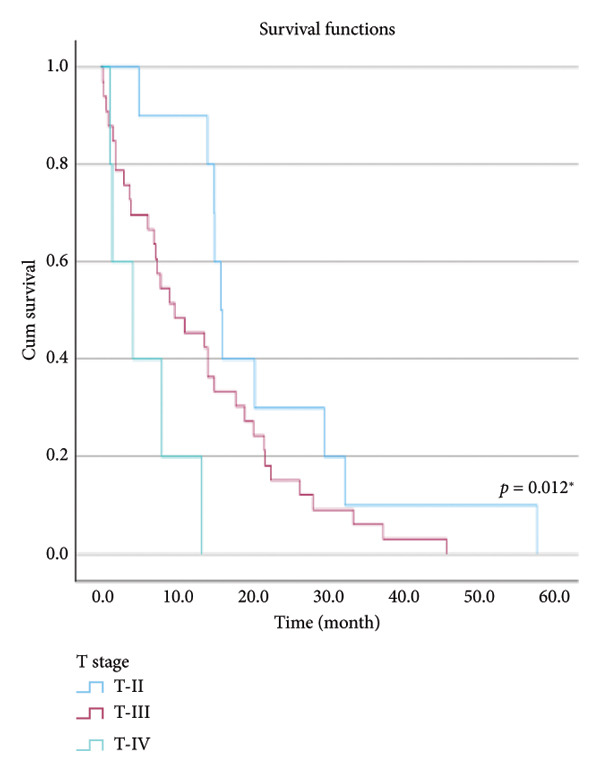
(g)

**Figure:**
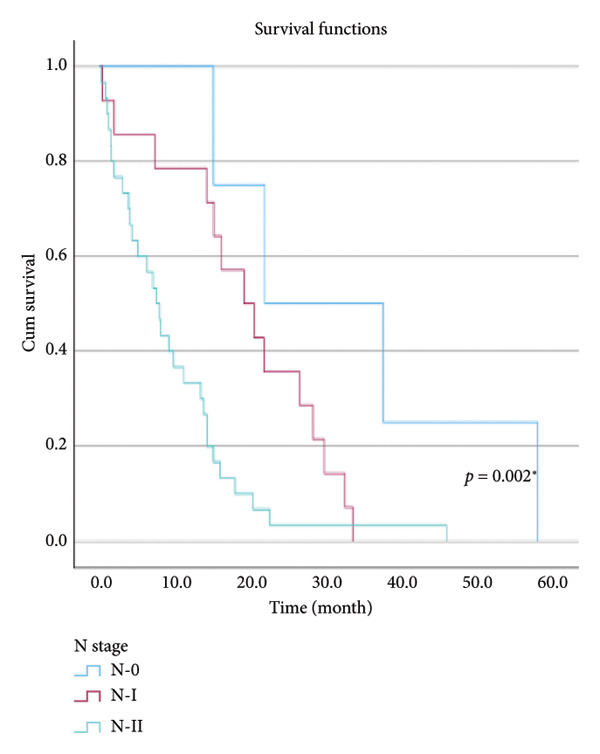
(h)

**Figure:**
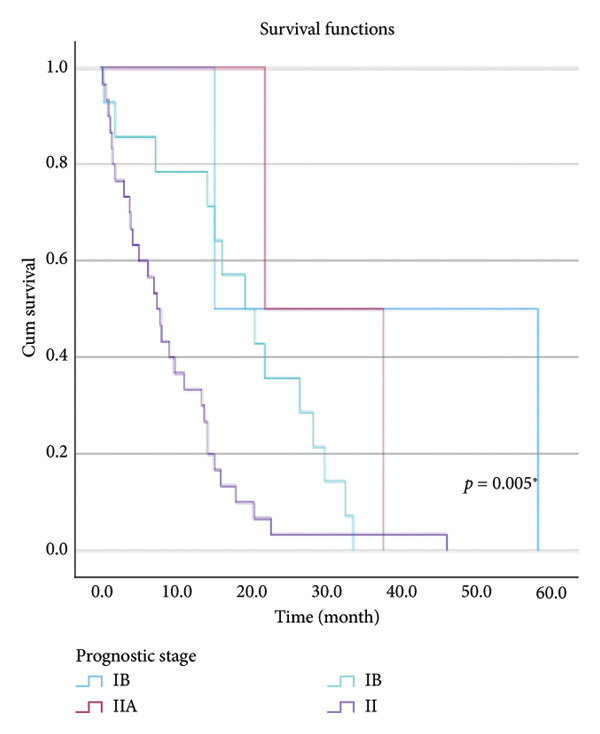
(i)

**Figure:**
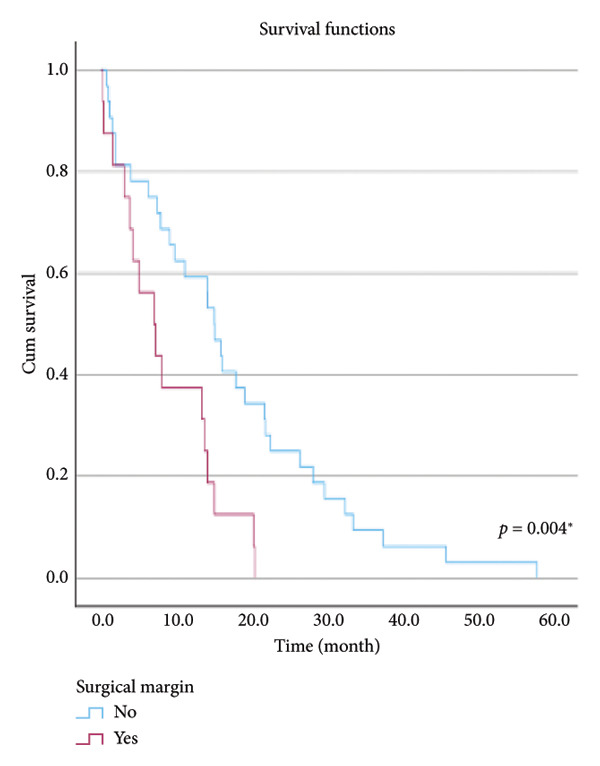
(j)

**Figure:**
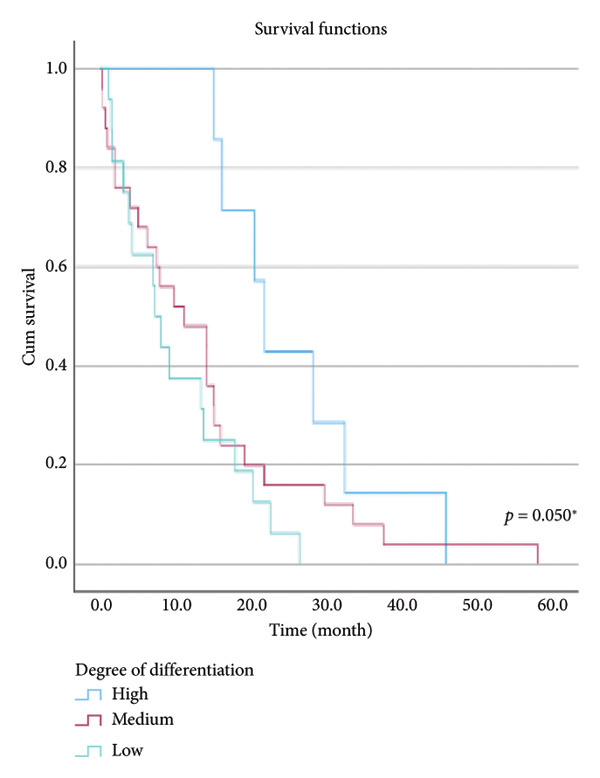
(k)

**Figure:**
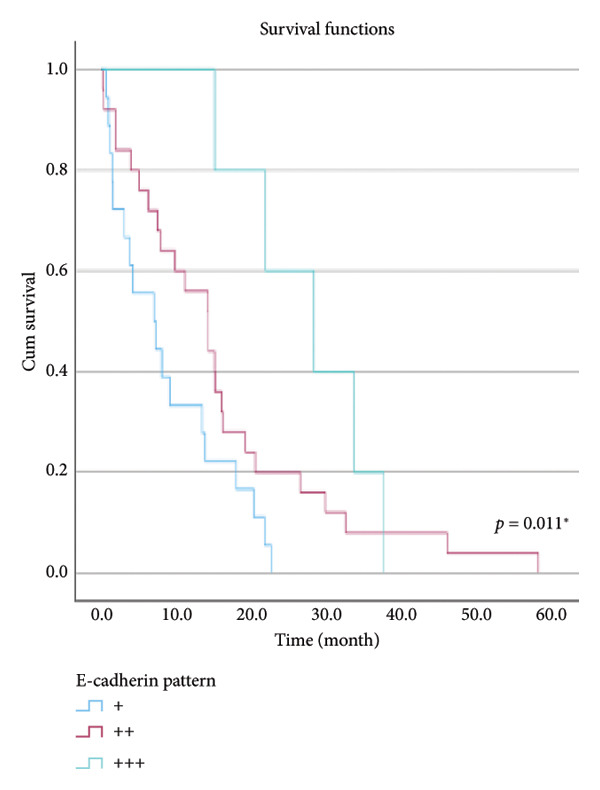
(l)

**Table 2:** Investigation of the effect of clinical parameters on the survival of the pancreatic cancer.

Parameters		Estimate	95% confidence interval		*p* value
			Lower bound	Upper bound	
Age	< 60	16.133	4.284	27.983	0.234
	> 60	9.133	1.305	16.961	

Gender	Male	14.200	7.065	21.335	0.822
	Female	11.133	2.212	20.055	

Tumor localization	Head	15.526	11.427	19.625	0.033^∗^
	Tail	8.329	1	15.818	
	Corpus	5.083	0	10.704	
	Uncinate	14.650	0.636	28.664	
	Head and uncinate	15.067	15.067	15.067	
	Head and corpus	9.800	9.800	9.800	
	Corpus and neck	57.933	57.933	57.933	

Perineural invasion	Negative	16.133	14.480	17.787	0.227
	Positive	11.133	3.641	18.626	

Lymphovascular invasion	Negative	39.867	4.456	75.277	0.046^∗^
	Positive	13.204	10.030	16.378	

T stage	II	15.967	14.366	17.568	0.012^∗^
	III	9.800	2.747	16.853	
	IV	4.267	0	10.207	

N stage	0	21.800	0	43.785	0.002^∗^
	I	19.100	11.216	26.984	
	II	7.500	5.085	9.915	

Prognostic stage	IB	36.500	0	78.509	0.005^∗^
	IIA	29.650	14.264	45.036	
	IIB	19.033	13.449	24.618	
	III	9.611	6.293	12.930	

Surgical margin	Negative	15.067	12.572	17.561	0.004^∗^
	Positive	7.033	2.656	11.411	

Degree of differentiation	High	21.800	18.293	25.307	0.050^∗^
	Medium	11.133	1	21.306	
	Low	7.267	5.241	9.292	

E‐cadherin pattern	Low (+)	7.033	1	13.270	0.011^∗^
	Medium (++)	14.200	9.227	19.173	
	High (+++)	28.200	14.459	41.941	

Tumor budding	No	21.700	17.663	25.737	< 0.001^∗∗^
	Yes	7.033	1.945	12.122	

^∗^ *p* < 0.05 is statistically significant.

^∗∗^ *p* < 0.001 is statistically significant.

In the univariate analysis (Figure 5(a)), several variables demonstrated a significant association with survival outcomes in the patient cohort. Tumor budding showed the strongest association with survival, with an HR of 7.834 (95% CI: 3.434–17.872, *p* < 0.001). Additionally, N stage (HR: 2.349, 95% CI: 1.435–3.847, *p* = 0.001), T stage (HR: 2.157, 95% CI: 1.189–3.916, *p* = 0.011), surgical margin (HR: 2.564, 95% CI: 1.317–4.992, *p* = 0.006), degree of differentiation (HR: 1.692, 95% CI: 1.098–2.608, *p* = 0.017), E‐cadherin pattern (HR: 0.492, 95% CI: 0.304–0.796, *p* = 0.004), and prognostic stage (HR: 2.138, 95% CI: 1.353–3.380, *p* = 0.001) were statistically significant. In the multivariate analysis (Figure 5(b)), tumor budding remained an independent prognostic factor for survival, with an HR of 6.594 (95% CI: 1.825–23.818, *p* = 0.004). Other variables, including T stage, N stage, surgical margin, and E‐cadherin pattern, did not retain statistical significance in the multivariate model. These findings highlight the critical role of tumor budding as a robust prognostic marker in the analyzed patient cohort.

Figure 5Prognostic factors associated with survival outcomes in the patient cohort. (a) Univariate analysis of clinical and pathological variables. (b) Multivariate analysis showing tumor budding as an independent prognostic factor for survival.

**Figure:**
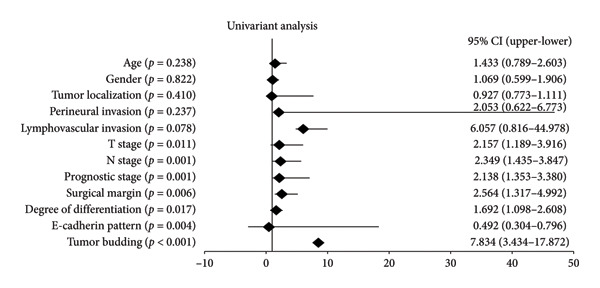
(a)

**Figure:**
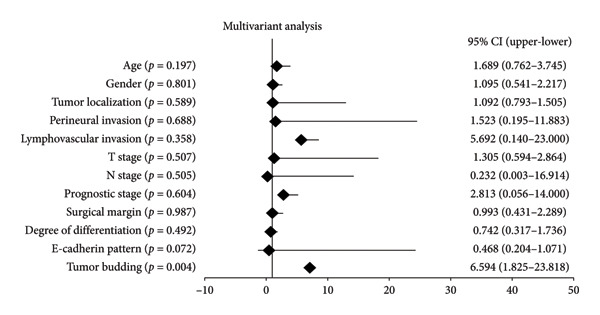
(b)

Subgroup analyses were performed based on the positivity of tumor buds, and factors affecting survival were examined by creating a Cox regression model for each subgroup in a multivariate model (Table 3). The model was created using survival time as the dependent variable and clinical parameters such as age, tumor stage, and E‐cadherin expression as independent variables. According to the subgroup analysis, a statistically significant association was identified in the tumor budding subgroup for E‐cadherin expression positivity (HR: 0.026, 95% CI: 0.002–0.324, *p* = 0.005), tumor stage (HR: 3.306, 95% CI: 1.037–10.538, *p* = 0.043), and differentiation grade (HR: 0.029, 95% CI: 0.002–0.432, *p* = 0.010). In the subgroup without tumor budding, significant associations were found with parameters such as localization, nodal stage, prognostic stage, and surgical margins.

**Table 3:** Cox regression analysis evaluation according to tumor budding status in subgroups.

Parameters	Budding	Exp (B)	95.0% CI for exp (B)		*p* value
			Lower	Upper	
Age	No	1.767	0.399	7.832	0.454
	Yes	1.207	0.434	3.359	0.718

Gender	No	0.342	0.072	1.629	0.178
	Yes	1.234	0.464	3.279	0.673

Tumor localization	No	2.201	1.185	4.086	0.012^∗^
	Yes	0.872	0.609	1.250	0.456

Perineural invasion	No	0.145	0.016	1.324	0.087
	Yes	—	—	—	—

Lymphovascular invasion	No	16.912	0.206	38.388	0.209
	Yes	—	—	—	—

T stage	No	0.582	0.099	3.146	0.549
	Yes	3.306	1.037	10.538	0.043^∗^

N stage	No	0.002	0	0.61	0.033^∗^
	Yes	0.321	0.048	2.150	0.241

Prognostic stage	No	58.588	1.708	30.364	0.030^∗^
	Yes	—	—	—	—

Surgical margin	No	39.398	3.922	75.364	0.002^∗^
	Yes	0.474	0.125	1.791	0.271

Degree of differentiation	No	3.371	1.105	10.284	0.053
	Yes	0.029	0.002	0.432	0.010^∗^

E‐cadherin pattern	No	0.81	0.125	5.233	0.825
	Yes	0.026	0.002	0.324	0.005^∗^

^∗^ *p* < 0.05 is statistically significant.

## 4. Discussion

Despite advances in the molecular analysis of cancer, histomorphologic assessment remains an important tool for the classification of malignant lesions. Tumor budding, which is recognized as a poor prognostic factor in colorectal carcinoma, is also gaining attention as a prognostic indicator in other gastrointestinal malignancies [15]. A meta‐analysis investigating tumor budding in gastrointestinal tumors other than colon carcinoma emphasized its importance as a prognostic biomarker in pancreatic cancer [16]. The aim of our study was to contribute to the literature by highlighting the importance of tumor budding as a prognostic factor in PDAC, similar to colon cancer. Furthermore, given the epidemiologic differences in PDAC, we argue for further country‐specific studies. This study is one of the few in the literature focusing on Turkish PDAC cases.

Our study aimed to investigate the prognostic significance of tumor budding in a cohort of Turkish patients diagnosed with PDAC, and to underscore the importance of including tumor budding as a prognostic factor in clinical guidelines. In addition, the relationship between tumor budding and E‐cadherin expression was investigated, and its contribution to clinical outcomes was evaluated. In this study, the clinical parameters of 76 patients with pancreatic cancer were analyzed. Of these, 56.6% were over 60 years old and 53.9% were male. In 76.3% of cases, the tumor was localized in the pancreatic head. Perineural invasion was found in 85.5% and lymphatic invasion in 88.2% of patients. In terms of T stage, 63.2% were in stage III and 48.7% in nodal stage II. Prognostically, 48.7% of patients were in stage III and 22.4 had a positive surgical margin. Tumor budding was found in 42.1% of patients, which was significantly associated with perineural and lymphatic invasion, advanced tumor stage, poor differentiation, and reduced E‐cadherin expression. Kaplan–Meier analysis revealed that positive tumor budding, advanced tumor stage, poor differentiation, and positive surgical margins as independent prognostic factors negatively affected survival.

In a separate study investigating the association between tumor budding and EMT in 50 PDAC patients after resection, high‐grade budding was found in 56% of cases and was identified as an independent negative prognostic factor, independent of tumor size, resection margin status, nodal involvement, and vascular invasion [17]. In our multivariate subgroup analyses, a significant statistical correlation was found between the negativity of the surgical margin and tumor spread. Tumor budding is highlighted as a marker of poor prognosis in pancreatic cancer. In a study of 120 PDAC patients, tumor budding was assessed using the ITBCC criteria, highlighting its importance as a parameter for overall survival and progression‐free survival [18]. In our cohort, approximately 25% of patients showed high‐grade loss of E‐cadherin expression, with over 80% of these patients also showing tumor budding. Subgroup analyses revealed a significant statistical correlation between E‐cadherin loss and tumor budding, underlining its crucial role as a prognostic biomarker. In patients with pancreatic head involvement, tumor budding was associated with lymph node ratio and E‐cadherin expression, with multivariate analyses showing reduced survival [19]. In our study, tumors of the pancreatic head accounted for the majority of cases and showed better survival compared to other subgroups. Subgroup analyses showed better survival in patients without tumor budding. Survival was significantly lower in patients with positive tumor budding. These results underline the importance of tumor biology in determining prognostic factors that influence survival.

In the prospective, multicenter CONKO‐001 study involving 173 PDAC patients, tumor budding was identified as a factor associated with tumor stage and shortened overall survival [20]. Our results also indicate a significant association between tumor stage and survival, with survival shortened to up to 4 months in stage IV patients. Subgroup analyses also showed that tumor budding significantly shortened survival in stage IV patients. In a study examining the frequency and prognostic significance of tumor budding in 117 PDAC cases, high‐grade tumor budding was observed in 70.3% of cases and was associated with T stage, lymphatic invasion, and decreased survival [21]. In our study, univariate analyses also revealed statistically significant associations between T stage, lymphatic invasion, and survival. In a study examining tumor budding in 75 PDAC patients using the ITBCC criteria, tumor budding was observed in 44% of cases and was significantly associated with invasion and shorter survival [22]. In our study, we also found statistically significant associations between survival and lymphatic invasion, T stage, N stage, and prognostic stage. In addition, multivariate subgroup analyses revealed a significant association between T stage and N stage. In another study of 130 consecutive PDAC patients, it was reported that patients with tumor budding had a twofold higher mortality rate [23]. In our cohort, the median survival time in patients with tumor budding was approximately 7 months compared to 21 months in patients without tumor budding, indicating a threefold reduction in survival time and underscoring the prognostic significance of tumor budding.

In a study on tumor budding and perineural invasion in PDAC, no association was found between budding and perineural invasion, but an association with lymph node metastases was found [24]. In contrast, our chi‐square analysis found a significant association between tumor budding and perineural invasion, but this was not significant as a prognostic factor. This finding contributes to the literature and emphasizes the need for further comprehensive studies. In a study examining tumor budding in 192 PDAC cases, it was found in 87.5% of patients and was associated with lower survival, tumor stage, grade, size, nodal status, lymphatic invasion, and perineural invasion [25]. Our results are consistent with these findings and will be further refined by subgroup analyses.

In slowly progressing pancreatic neuroendocrine tumors, tumor budding has been reported as a valuable parameter for the assessment of postoperative liver metastasis risk [26]. Our study focused on nonmetastatic PDAC cases, and distant metastases were not investigated. We plan future studies on the impact of tumor budding in long‐term metastatic PDAC cases. A meta‐analysis of 613 PDAC cases found an association between tumor budding and disease‐related mortality and recurrence [27]. However, as no recurrences were observed in our patient cohort, we could not evaluate the impact of tumor budding on recurrence. Finally, tumor budding has been reported to significantly shorten survival in PDAC cases resected after chemoradiotherapy, regardless of treatment [28]. Our cohort consisted of untreated PDAC patients, so we investigated the effects of treatment on tumor budding. Future studies will investigate the effects of chemotherapy and radiotherapy on the survival of patients with tumor budding.

The findings highlight the impact of tumor stage and biological markers on survival outcomes, corroborating previous studies that associate advanced tumor stages with shorter survival durations. However, limitations such as small sample sizes in certain groups and a high proportion of censored cases may restrict the reliability of the analyses. This study emphasizes the importance of considering tumor stage and biological markers in clinical decision‐making processes and recommends replicating such analyses with larger sample sizes.

## 5. Conclusion

This study underscores the significant prognostic role of tumor budding in PDAC and emphasizes its association with poor clinical outcomes, including advanced tumor stage, perineural and lymphatic invasion, poor differentiation, and reduced survival. The strong correlation between tumor budding and E‐cadherin loss underscores its importance as a biomarker. Patients with tumor budding had significantly shorter survival, highlighting its value as an independent prognostic factor. While our findings are consistent with the existing literature, the study also highlights the need for larger, multicenter studies to validate these associations and investigate the impact of tumor budding in metastatic PDAC and in the context of post‐treatment. These findings highlight the potential of incorporating tumor budding into clinical guidelines to improve prognostic accuracy and inform treatment strategies.

## Ethics Statement

The study was approved by the Clinical Research Ethics Committee of Bagcilar Training and Research Hospital and conducted by the Helsinki Declaration (Date: 2025.02.04/No: 021).

## Conflicts of Interest

The authors declare no conflicts of interest.

## Author Contributions

Tevhide Bilgen Özcan contributed to conceptualization, writing, review and editing, and supervision. Formal analysis was performed by Tevhide Bilgen Özcan and Esra Pasaoglu. Osman Bilgin Gülçiçek provided resources.

## Funding

This research received no external funding.

## Data Availability

The datasets generated and/or analyzed during the current study are not publicly available due to ethical and privacy restrictions but are available from the corresponding author upon reasonable request.
